# Enhancing Women’s Well-Being: The Role of Psychological Capital and Perceived Gender Equity, With Social Support as a Moderator and Commitment as a Mediator

**DOI:** 10.3389/fpsyg.2019.01377

**Published:** 2019-06-19

**Authors:** Sonam Chawla, Radha R. Sharma

**Affiliations:** Organizational Behavior, Management Development Institute, Gurgaon, India

**Keywords:** commitment, gender equity, Indian study, psychological capital, social support, well-being, women’s well-being

## Abstract

The study aims to determine the role of psychological capital and perceived gender equity on employee well-being, particularly women, and assess if commitment mediates and social support moderates the relationships between psychological capital, perceived gender equity, and well-being. A personal survey method was employed for data collection using standardized measures from a representative sample of 433 managers (201 women and 233 men) from private sector companies in India. The findings revealed that perceived gender equity in the workplace positively impacts employee well-being for both men and women, with the greater impact being on women’s well-being. To Facilitate employee well-being, organizations can leverage the strengths of psychological capital through training interventions and can promote perceived gender equity through appropriate policies and practices. This bridges the knowledge gap in developing and utilizing psychological capital to enhance employee well-being, especially for women, who are under pressure due to their demanding multiple roles at work and home.

## Introduction

Organizations, in a knowledge- and information-driven economy, are realizing the importance of human capital in gaining and sustaining a competitive edge (Williams et al., 2016). With increasing competition and the paucity of trained human resources (Frank et al., 2004), organizations are focusing more than ever before on attracting and retaining talent. In addition, employees increasingly expect organizations to play an active role in supporting their well-being, which strengthens the organizations’ positions as attractive employers (Martin et al., 2005). Furthermore, employee well-being is important to organizations due to its connection to higher productivity, dependability, and overall work quality (Diener et al., 2002). Employee well-being has been found to be related to their making “personal sacrifices for the client,” going beyond the call of duty and expectation (O’Donohoe and Turley, 2006). Though economic developments have increased the employment opportunities for women, their happiness and well-being have decreased since the 1970s compared to men (Stevenson and Wolfers, 2009). One probable reason is that women’s participation in the workforce has not been accompanied by a shift in their household responsibilities, thus they work a “second shift” (Hochschild and Machung, 1989; Aguiar and Hurst, 2007). Thus, the volatile economic conditions and increasing work pressures have made employee well-being a pivotal subject of investigation, the strain being more pronounced for women (Sharma and Sharma, 2015). The purpose of the study is to investigate the influence of psychological capital (PsyCap) and perceived gender equity (PGE) on women’s well-being. We also investigated commitment as a mediator and social support as a moderator between PsyCap and PGE on well-being. Drawing on the data pertaining to 433 employees (men and women) in service sector companies in India, this study conducted an empirical analysis using structural equation modeling to find the role of PsyCap and PGE in women’s well-being.

Scarcity of talent has led organizations to realize the importance of directing their efforts toward attracting, advancing, and retaining women in the workforce (Kochan et al., 2003; Tatli et al., 2013). Also, there is an increasing realization that gender diversity, if managed well, would yield dividends in enhancing performance (Kochan et al., 2003). Research evidence suggests that both men and women enter the workforce at comparable levels; however, their career paths begin to diverge after a point as women continue to face challenges while climbing the management ladder (Bowling and Beehr, 2006; Riccucci, 2009; Davidson, 2012). Consequently, a number of women, dissatisfied with their treatment at work and the lack of advancement opportunities, leave their corporate careers mid-way (Townsend, 1997; Kottke and Agars, 2005). According to Catalyst (2016), in Standard and Poor’s (S&P) 500 companies, there have been 44.7% total female employees, of which 26.5% were at mid-level managerial positions and only 5.2% at the CEO level. Whereas in India, 19% of employees in senior management were women (Catalyst, 2016) and 7% were women at the CEO/Managing Director level. According to the World Bank Report (2016), women’s participation in the Indian labor force continues to fall from 34.8 to 26.7%, which ought to be a cause for concern for these organizations. Societal beliefs and expectations make working Indian women more vulnerable to stress, as they are expected to not only play dual roles at home and work but also to perform well in the workplace (Naqvi, 2011; Chawla and Sharma, 2016). The Nielsen Survey reports that working Indian women are the most stressed in the world, as 87% of working Indian women reported that they felt stressed, anxious, and unenthusiastic, while 82% said that they had no time to relax. Also, 55% of working Indian women experienced stress because of workplace bias and gender discrimination. This trade-off between household responsibilities and work demands, as well as the resultant stress, make them leave their jobs early on (Radhakrishnan, 2011). The attrition of female employees along the career track entails considerable loss to the organization due to investment in their potential development. Taylor (2002) suggested that relationships in the workplace, fair treatment, and work environment may be more important to the well-being of women than that of men, as women are likely to experience both positive and negative emotions more intensely than men (Fujita et al., 1991). The reasons mentioned above make a compelling case for studying the determinants of well-being in female employees.

Psychological capital, which finds its roots in the field of positive organizational behavior (POB), comprises of an individual’s positive psychological resources, namely hope, optimism, self-efficacy, and resilience (Luthans et al., 2007). It has been found to have an inherent ability to enhance well-being (Luthans et al., 2010). Though Avey et al. (2010) have found preliminary evidence linking PsyCap and well-being, there have been fewer studies investigating the mechanisms through which this relationship may be fostered (Williams et al., 2016). There is a research gap about how this relationship varies for different groups and how PsyCap can be developed to help women enhance their well-being.

Furthermore, fairness and equity in the workplace are known to be important inputs to an individual’s well-being (Wilkinson and Pickett, 2010; David et al., 2012), but have been rarely considered by prior studies except in a few contexts like fairness perceptions of employee feedback (Sparr and Sonnentag, 2008) and organizational justice (Lawson et al., 2009), and rarely in the context of female employees (Burke et al., 2009; Sharma and Sharma, 2015). It is established that discrimination in the workplace affects the well-being of employees (Schmitt et al., 2002).

Review of the extant literature yielded a few studies on organizational and personal factors adversely affecting working women’s well-being, but there is a paucity of studies focusing on how to enhance their well-being. In this study, we have tried to determine how organizations can contribute to the well-being of employees, especially women, through a gender-equitable environment and by leveraging their positive psychological resources. We investigate this through the composite construct of PGE encompassing organizational policies, practices, and the environment, leading to gender-equitable perceptions among the employees (Sharma Radha, 2013; Sharma and Sharma, 2015) and PsyCap that affects their well-being. The study also investigates the role of commitment and social support as intervening variables between PGE, PsyCap, and well-being, and suggests measures for enhancing well-being.

## Theoretical Background and Hypotheses

### Well-Being

Well-being at work has been defined in a variety of ways in the extant literature (Lyubomirsky, 2007; Page and Vella-Brodrick, 2009; Fisher, 2010; Sharma and Sharma, 2015). For the present study, well-being has been considered as subjective well-being (SWB) (Diener, 1984), which is the dominant conception in the literature (David et al., 2013). SWB is how people evaluate their lives in terms of affect (how we feel) and cognitive (how we think) components (Diener et al., 1999, 2016). The affective component is associated with emotions and the experience an individual has of momentary events in his/her life. The cognitive component consists of life satisfaction concerning how individuals perceive their lives and referring to the discrepancy between their present situation and what they think is ideal for them. The settings in which one works – support at work, the nature of interactions and relationships with others, the work environment (Barling and Frone, 2004), and job characteristics (Warr, 2013) – all affect the well-being of an employee. SWB has been found to predict health and longevity of employment (Liu et al., 2010; Diener and Chan, 2011; Kuykendall and Tay, 2015; Tay et al., 2015), employees going beyond the call of duty for customers, lower turnover, and a lower intention of quitting (Podsakoff et al., 2007). Also, well-performing firms encourage positive affect (PA) in employees (Losada and Heaphy, 2004; O’Donohoe and Turley, 2006). Furthermore, we used the construct of SWB for this study, as it encompasses not only work but also non-work facets that affect work, which have recently been recognized as important aspects in evaluating an employee’s well-being (Diener et al., 2017).

Volatile economic conditions have made employees insecure about their employment status, future career prospects, and long-term financial security (Guest, 2017). Also, while technology has been a boon with respect to the automation of routine tasks, access to information, and flexible working, it has also led to “work-home interference” (Derks et al., 2014), decreased autonomy, and increased work demands (Felstead et al., 2015). This has reduced the well-being of employees in organizations (Guest, 2017), calling for investigation.

#### Well-Being and Women

Women’s joining the workforce has not been accompanied by a change in their household responsibilities (Aguiar and Hurst, 2007), resulting in additional roles for working women. The stress hypothesis theory of multiple roles (Goode, 1960) suggests an individual’s well-being reduces when an additional role is added to the societal role that already includes many demands and hassles. Working women experience the same while meeting the demands of high performance at work as well as managing children and the household. Krueger (2007) estimated that women became hedonically worse off compared to men between 1966 and 2005. Most of the previous studies on women’s well-being have focused on physical health, such as cardiovascular diseases (Ebi-Kryston et al., 1990; Pavalko and Smith, 1999), and only a few have focused on their psychological well-being (Kopp and Ruzicka, 1993; Martire et al., 2000; Sharma and Sharma, 2015). Hence, the present study focuses on psychological capital.

### Psychological Capital and Well-Being

Psychological capital has its origins in positive psychology (Seligman, 1998) and positive organizational behavior (POB) (Luthans, 2002; Cameron et al., 2003; Wright, 2003). PsyCap is defined as the following:

An individual’s positive psychological state of development characterized by: (i) having confidence (efficacy) to take on and put in the necessary effort to succeed at challenging tasks; (ii) making a positive attribution (optimism) about succeeding now and in the future; (iii) persevering toward goals and, when necessary, redirecting paths to goals (hope) in order to succeed; and (iv) when beset by problems and adversity, sustaining and bouncing back, and even beyond (resilience) to attain success (Luthans et al., 2007, p. 3).

PsyCap has the ability to influence people’s positive appraisals and understandings of situations, which can, in turn, influence their well-being (Avey et al., 2010). Youssef and Luthans (2013) stated that the cognitive and affective mechanisms of PsyCap can contribute to the well-being of employees. People with high psychological capital can, therefore, appraise demanding and difficult situations positively with a positive state of mind and emotions. Moreover, in comparison to other “trait-like” psychological resources, like personality, character, intelligence, and temperament, PsyCap is more “state-like,” which is malleable and open to development and interventions (Luthans et al., 2007). “Trait-like” characteristics are more static, largely genetically determined, and therefore, difficult to change (Luthans and Avolio, 2014). Thereby, PsyCap has been selected as a study variable.

#### PsyCap and Well-Being in Women

Working women play multiple roles at work and at home while being constrained for time, which may contribute to health problems, stress, anxiety, and other negative outcomes (Gjerdingen et al., 2001). Each of the four dimensions of PsyCap (hope, efficacy, resilience, and optimism) has the potential of playing an important role to help a female manager overcome the challenges at work and in managing stress. Hope (Snyder et al., 1991) signifies the willpower for finding alternate paths to achieve goals; women with hope can expect to succeed given the constant redirection of career paths that takes place in a woman’s professional life (Mainiero and Sullivan, 2005). Self-efficacy (Bandura, 1997) is the confidence and belief in one’s own ability to overcome challenging situations (Newman et al., 2014). It is pivotal for women to feel confident about the skills, abilities, and knowledge that they bring to the workplace, considering the societal skepticism about their efforts and abilities to perform. Resilience is the capacity to bounce back from adverse conditions, failure, and conflict (Luthans, 2002). When one considers the common setbacks faced by women in the workplace (like gender bias and attribution of their success to non-merit factors), resilience becomes an important resource for a woman to grow in her career without becoming dejected. Lastly, optimism (Seligman, 1998) is the ability to attribute positive events to internal and lasting causes (Youssef and Luthans, 2013) and negative events to temporary and external situations. In the case of working women, optimism plays an important role in negative situations that they face in their organizations. Women at work, therefore, might benefit with the development of PsyCap by lowering the experience of negative emotions and enhancing well-being. According to Combs et al. (2012), PsyCap might be of greater benefit to marginalized groups in the workplace, given that it will provide them with the confidence to seek much-needed support and advice from others in the workplace. Few studies have focused on the relationship between PsyCap and subjective well-being in an organizational setting (Nguyen and Nguyen, 2012; Baron et al., 2013), and there is some initial evidence that PsyCap positively impacts the subjective well-being of individuals over time (Avey et al., 2010; Culbertson et al., 2010; Luthans et al., 2013). PsyCap, with a potential to protect and defend the well-being of women at work, has never been studied in relation to enhancing women’s well-being. Thus, we hypothesize the following:

Hypothesis 1a. PsyCap has a positive and significant relation to well-being.
Hypothesis 1b. There is a significant difference between the relation of PsyCap to well-being concerning men vs. women.
Hypothesis 1c. The positive effect of PsyCap on well-being is stronger for women than for men.

### Perceived Gender Equity and Well-Being

In the environment-centered happiness perspective, David et al. (2013) identified equity at the workplace as one of the important factors affecting well-being. Perceptions of fairness at work are important inputs to well-being at work. The equity theory (Adams, 1965) also confirms that people need to feel that their inputs are equal to their perceived outputs, in order to feel fairly treated. Therefore, the setting in which one works, work policies, treatment, and interactions with other people all contribute to a person’s well-being (Caza and Wrzesniewski, 2013). Any perceived imbalance would affect the well-being of employees. Furthermore, social identity theory (Tajfel and Turner, 1986) stresses the importance of inclusion for well-being and predicts that rejection from important social groups can be painful.

Women have been facing discrimination and unfair treatment at work due to organizational policies and processes (Kanter, 1993). The gendered perceptions (stereotypes and gender roles) at the workplace further create injustice for women (Chawla and Sharma, 2016). The aforementioned causes lead to a feeling of discrimination and stress for working women, which in turn, negatively affects their well-being. Also, Sharma and Sharma (2015) established a negative relationship in their study between perceived gender equity and burnout in the workplace for both men and women. Though organizational policies exist for facilitating women’s advancement and fair treatment, they remain a mere eyewash because of the gendered perception supervisors and peers have and their inability to put these into practice (Eagly and Carli, 2007). Consequently, equitable employment opportunities play a crucial role in balancing an employee’s private and professional life, and thus are expected to help women maintain the work/life balance. This can be possible if organizations formulate policies involving the reconciliation of work and care, temporary withdrawal from work, leave arrangement, and professional support (Burgess et al., 2007), and also provide an environment to avail of such policies. The construct that appropriately explains this in the literature is PGE. The construct of PGE in the workplace was evolved in 2013 by Sharma Radha, who also developed and standardized a measure for it. PGE in the workplace is defined as “employees’ positive perception of equal opportunity in recruitment, training and development, compensation, career progression, dignified treatment and professional respect through the organizational policies, practices and environment” (Sharma Radha, 2013). The construct is comprised of three dimensions: “equity perception through organizational policies,” “equity perception through organizational practices,” and “equity perception through organizational environment” (Sharma and Sharma, 2015). Therefore, the construct considers not only the presence of equitable policies but also their implementation and a conducive environment in which to implement them. PGE has been found to be positively related to work engagement and general satisfaction with life and work, and negatively related to burnout (Sharma and Sharma, 2015). We therefore hypothesize the following:

Hypothesis 2a. PGE has a positive and significant relation to well-being.
Hypothesis 2b. There is a significant difference between the positive effect of PGE and well-being concerning men vs. women in the workplace.
Hypothesis 2c. The positive effect of PGE on well-being is stronger for women than for men.

### Commitment

Organizational commitment refers to the strength with which an employee binds oneself to the organization and displays behaviors that are valuable to the organization (Allen and Meyer, 1990; Meyer et al., 2006). As economic uncertainty continues to plague the world, the commitment of each employee to their organization is essential to both the employee and the organization for positivity and professionalism. Companies today face a huge challenge in maintaining employee commitment, particularly for minorities and women (Ensher et al., 2001). Commitment has been classified as either affective, normative, or continuance commitment (Meyer and Allen, 1984). Affective commitment is said to have the maximum effect on organizationally desired behavior (Meyer and Allen, 1991). In addition to feeling satisfied, Allen and Meyer (1990) established that equity is an important antecedent to affective commitment. Also, an employee’s perception of equity or inequity would lead to higher or lower affective commitment, respectively (Tang and Sarsfield-Baldwin, 1996; Ngo et al., 2014).

PsyCap has been studied in the context of sales organizations as a potential tool for increasing organizational commitment (Schulz et al., 2014). Luthans and Jensen (2005) established the positive relationship between nurses’ self-reported positive PsyCap and their “intentions to stay,” as well as their supervisors’ ratings of their commitment to the organization’s mission statement. Furthermore, social exchange theory states that when an employee faces unjustified barriers and unfair treatment at the workplace, they lower their commitment to the organization (Graves and Powell, 1994; Auster, 2001). Research has shown that affective commitment is positively related to mental health (Probst, 2003; Grawitch et al., 2007), positive affect (Thoresen et al., 2003), zest, enthusiasm, and vitality (Lester et al., 2010). Scholars have also found negative relations between affective commitment and measures of strain, such as psychosomatic symptoms and physical health complaints (Richardsen et al., 2006; Wegge et al., 2006), negative affect (Thoresen et al., 2003), and burnout (Hakanen et al., 2006; Grawitch et al., 2007). Attempts to develop theories (Meyer and Maltin, 2010; Meyer et al., 2012) have used the framework of self-determination theory, positing that commitment’s links with well-being can be explained, at least in part, as an indicator that employees’ life satisfaction is being met at work. Therefore, we hypothesize the following:

Hypothesis 3. The relationship between PsyCap and well-being will be mediated by commitment.
Hypothesis 4. The relationship between PGE and well-being will be mediated by commitment.

### Social Support

High levels of work pressures, along with the increasing responsibilities of childcare and elder care at home, create competing demands between work and family roles that in turn have an impact on the overall well-being of employees (Drummond et al., 2017). These challenges and stress become more pronounced for women, because they assume the role of primary caregivers. Social support is one of the resources that buffers the strain and stress caused by such increasing demands (Drummond et al., 2017). Social Support is frequently linked to positive psychological and physical outcomes. It acts as an environmental coping resource, which interacts with stress, offers a buffer for negative feelings and enhances positive feelings (Sackey and Sanda, 2011); on the other hand, the lack of social support leads to negative psychological states, such as anxiety, helplessness, and depression (Cohen and Wills, 1985). In the extant literature, social support has been studied as work-based or family based support, with most of the studies in organizational settings focusing on support provided within the organization (Scott-Ladd et al., 2010). There are differences in requirement of social support for men and women (Sackey and Sanda, 2011). Especially in the case of women, social support, both work and family related, may play an important role in ensuring their well-being. Therefore, for the purpose of this study, we have defined social support as “the help, encouragement, assistance and other benefits in managing responsibilities at home and work, as received from spouse, extended family, friends, supervisor and coworkers” (Marcinkus et al., 2007). Based on the foregoing discussion, we hypothesize that:

Hypothesis 5a. Social Support moderates the relationship between PsyCap and well-being, such that high social support leads to greater well-being.
Hypothesis 5b. There is a significant difference between the moderating role of social support in PsyCap and well-being among men and women.
Hypothesis 5c. The moderating effect of social support between PsyCap and well-being is stronger for women than men.
Hypothesis 6a. Social Support moderates the relationship between PGE and well-being, such that high social support leads to greater well-being.
Hypothesis 6b. There is a significant difference between the moderating role of social support in PGE and well-being among men and women.
Hypothesis 6c. The moderating effect of social support between PGE and well-being is stronger for women than men.

**FIGURE 1:**
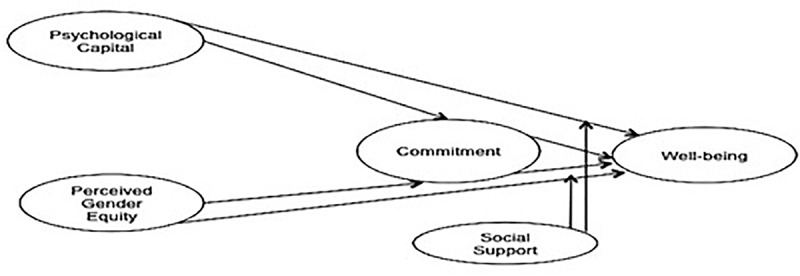
Conceptual model.

The study’s conceptual model is presented in Figure 1.

## Materials and Methods

### Participants and Procedures

The sample of participants for the study was drawn employing a stratified random sampling technique. The companies were selected from the list of the top 100 companies ranked in the Great Place To Work^®^, India, 2016, which among other criteria takes into account trust, pride, and the extent to which employees expect to be treated fairly^1^. From these private sector companies, the employees (men and women) from the middle and senior levels of management were selected as a sample.

The companies’ human resource departments were approached for their consent for data collection and were told the context of the study. The companies were selected from service industries comprising banking and financial services, IT/ITes, Telecom, consulting, and fast-moving consumer firms. All the companies were similar in their work culture and the type and number of employees. Since gender equity was considered to be a sensitive subject by the organizations, they agreed to participate in the survey on the condition of anonymity. After the consent of the HR managers, a personal survey method was adopted for data collection. The interested employees were assembled at a scheduled time. After briefing the participants about the purpose of the study and obtaining their informed consent, the questionnaires were distributed, and the respondents were requested to fill in the questionnaires with genuine responses. Multiple meetings were held at the 18 selected companies from which data were collected from 25–30 managers per company. A random sampling method was adopted to collect data from a representative sample of 449 employees. However, only 433 completed the measures, resulting in a response rate of 96.65% with 202 female respondents (46.7%) and 231 male respondents (53.3%). 20.4% respondents were from the banking/financial services industry, 20.1% from the IT/ITes industry, 16.7% from the telecom industry, 13.8% from the consulting industry, 12.4% from the retail industry, 11.1% from other industries, and 5.5% from the FMCG/consumer goods industry. The average age of the participants was 36.2 years, and the average work experience was 13.08 years.

The average time taken by the respondents was 15 min. This method, though time consuming, had the advantage of seeking informed consent, establishing rapport, standardizing the procedure and instructions, and clarifying doubts on the spot, resulting in a higher response rate (96.65%) compared to an impersonal mail survey. The data were collected with the objective of assessing the role of perceived gender equity and psychological capital in well-being for both men and women to study gender differences, if any.

### Measures

Standardized measures were used, and all the measures were tested for their reliability and validity on the study sample. These results are reported later.

#### Psychological Capital

Psychological Capital Questionnaire (PCQ-12) by Luthans et al. (2007) has been used to measure the four dimensions of PsyCap: hope, self-efficacy, resilience, and optimism. The scale has demonstrated reliability and validity in previous research (Avey et al., 2008). The scale is a 6-point Likert scale ranging from 1 (strongly disagree) to 6 (strongly agree). Its Cronbach’s alpha on the present study sample was 0.995

#### Perceived Gender Equity

The PGE scale by Sharma Radha (2013) is comprised of 29 items. Some of the sample items are “both males and females in the organization are selected for leadership roles” and “management’s organizational policy reflects commitment toward fair treatment of both male and female employees.” The ratings were made on a 6-point Likert scale ranging from 1 (*not at all true)* to 6 (*always true).* A high score indicates high perceived gender equity. The scale has been used in previous studies and has been found valid and reliable (Sharma and Sharma, 2015). Its Cronbach’s alpha on the study sample is 0.989.

#### Commitment

Employees’ affective commitment to the organization was assessed using Meyer and Allen’s (1997) 8-item measure of the Organizational Commitment Scale. Responses to each item were made on a 6-point scale with anchors labeled from 1 *(strongly disagree)* to 6 *(strongly agree).* Its Cronbach’s alpha on the study sample is 0.944.

#### Social Support

The social support received was measured using the social support scale developed by Marcinkus et al. (2007), which measures work-based social support and personal social support. It asked respondents to rate “how much do each of the following help you to balance your work and non-work activities”; the seven sources of support included: spouses, extended family, supervisors, and others. Respondents rated each source of support on a 6-point Likert scale ranging from “none” to “a great deal.” A high score indicates high social support. Its Cronbach’s alpha on the study sample is 0.933.

#### Well-Being

This was measured using the 20-item item Positive and Negative Affect Schedule (PANAS; Watson et al., 1988) and the 5-item Satisfaction With Life Scale (Diener et al., 1985). Participants rated all of the items on these two scales on a 6-point Likert scale, in which a score of 1 indicated slight or no experience of the emotion and a score of 6 reflected an intense experience of the emotion. The Satisfaction With Life Scale consists of five unidirectional attitude expressions. Participants rated their agreement with the expressions on a 6-point Likert scale ranging from 1 *(strongly disagree)* to 6 *(strongly agree).* A high score indicates the experience of more positive emotions and a higher satisfaction with life. Its Cronbach’s alpha on the study sample is 0.929.

#### Control Variables

Based on earlier studies, age, work experience, marital status, and the number of children were treated as control variables due to their possible confounding effect on the study variables.

## Results and Analysis

The results were analyzed for the total sample of 433 respondents and also for male and female respondents using the Statistical Package for the Social Sciences (IBM SPSS, version 21) and the Analysis of Moments Structure (AMOS), version 20.0. The analysis followed a four-step process: (1) descriptive analysis was carried out to determine the means and standard deviations (2) factorial invariance was established; as we were measuring constructs across two groups (men and women), it was first of all essential to validate that the factor structure and loadings were sufficiently equivalent across groups (Vandenberg and Lance, 2000) (3) scale analysis and measurement model analysis for each measurement instrument was completed through CFA to ensure its reliability and validity (Byrne, 2001) (4) path model analysis using structural equation modeling (SEM) was completed, and partial least squares techniques involving maximum likelihood estimation (MLE) were used. For comparing the differences between men and women for hypotheses 1a, 1b, 1c, 2a, 2b, 2c, 5a, 5b, 5c, 6a, 6b, and 6c, we carried out multi-group analysis (Steenkamp and Baumgartner, 1998).

### Basic Statistics and Correlations

Means, standard deviations, and correlations among variables are reported in Tables 1A–C. The correlation matrix was analyzed in the context of the hypothesized model. These indicate hypothesized positive relationships between PsyCap and commitment, PsyCap and social support, PsyCap and well-being, PGE and commitment, PGE and social support, and PGE and well-being, all of which are statistically significant (*p* < 0.01).

**Table 1A:** Means, standard deviations, and inter correlations among study variables.

	Mean	SD	1	2	3	4	5
Variables							
1. Psychological capital	4.1	1.474	1				
2. Perceived gender equity	4.11	1.44	0.484^∗∗^	1			
3. Commitment	3.746	1.409	0.749^∗∗^	0.642^∗∗^	1		
4. Social support	3.37	1.423	0.251^∗∗^	0.702^∗∗^	0.334^∗∗^	1	
5. Well-being	4.361	1.16	0.585^∗∗^	0.082^∗∗^	0.514^∗∗^	−0.056	1

*^∗∗^p < 0.01.*

**Table 1B:** Means, standard deviations, and inter correlations among study variables (Women).

	Mean	SD	1	2	3	4	5
Variables							
1. Psychological capital	4.42	1.406	1				
2. Perceived gender equity	4.1	1.43	0.491^∗∗^	1			
3. Commitment	4.11	1.44	0.846^∗∗^	0.735^∗∗^	1		
4. Social support	3.61	1.41	0.212^∗∗^	0.711^∗∗^	0.315^∗∗^	1	
5. Well-being	4.1	1.1	0.505^∗∗^	0.115^∗∗^	0.612^∗∗^	−0.042	1

*^∗∗^p < 0.01.*

**Table 1C:** Means, standard deviations, and inter correlations among study variables (Men).

	Mean	SD	1	2	3	4	5
Variables							
1. Psychological capital	3.8	1.51	1				
2. Perceived gender equity	4.12	1.45	0.487^∗∗^	1			
3. Commitment	3.38	1.35	0.672^∗∗^	0.549^∗∗^	1		
4. Social support	3.14	1.43	0.291^∗∗^	0.769^∗∗^	0.315^∗∗^	1	
5. Well-being	4.62	1.22	0.611^∗∗^	0.049^∗∗^	0.416^∗∗^	−0.073	1

*^∗∗^p < 0.01.*

### Factorial Invariance

A factorial invariance test was done to establish the equivalence of male and female data, in order to determine whether the instruments can be used and compared across the two groups (Vandenberg and Lance, 2000). In order to establish this, we compared two models that are specified by constraining (the constrained model) all the parameters for the two groups and by freely estimating (the unconstrained model) the parameters for the groups. For each model being compared, a chi-square fit statistic was iteratively estimated from the minimization of the difference between the model’s implied mean and covariance matrices and the observed mean and covariance matrices. The significance of whether increasingly restrictive models produce appreciable changes in chi-square values indicates that the measurement construct is not equivalent across different groups. In addition, if the difference between the comparative fit index (CFI) of the two models is greater than 0.01, we conclude that the measurement construct is not equivalent across different groups (Vandenberg and Lance, 2000). The chi-square values for the constrained and unconstrained models for the male and female respondents for all the study variables are summarized in Table 2. It can be observed that the difference between the chi-square values for the unconstrained models is not significant (*p* < 0.05) for the constructs. Furthermore, the difference in CFI values of two models is not greater than 0.01, thus implying that the measurement constructs are equivalent across the two groups (men and women).

**Table 2:** Establishing factorial invariance.

Variables	Type of model	Chi-Square value	*p*-value^∗^	Chi-Square difference	*p*-value^∗^	CFI
Psychological capital	U	168.33	0.14	24.41	0.08	0.991
	C	192.75	0.21			0.99
Perceived gender equity	U	663.19	0.27	22.05	0.28	0.967
	C	685.24	0.15			0.967
Commitment	U	18.07	0.11	1.63	0.95	0.998
	C	19.71	0.34			0.999
Social support	U	17.19	0.24	16.07	0.14	0.998
	C	33.19	0.17			0.994
Well-being	U	497.58	0.21	27.82	0.07	0.963
	C	525.41	0.25			0.962

*U is unconstrained model and C is constrained model. ^∗^p < 0.05.*

### Measurement Model Testing

Confirmatory factor analysis (CFA) was carried out to determine the distinctiveness of each of the measures used in the study and to verify the factor structure between the manifest and their underlying latent constructs. The individual measurement model was first assessed for each of the measures. The measurement model fit indices of these measures are reported in Table 3A. The overall model fit was assessed using various fit indices such as the chi-square (χ^2^) test, the goodness of fit (GFI), the Tucker-Lewis index (TLI), the comparative fit index (CFI), and root-mean square error of approximation (RMSEA). The values of the measurement fit indices obtained for each of the indices suggested acceptable model fit (Byrne, 2001; Hair et al., 2006), as reported in Table 3B. In order to strengthen the case for the relationships in the hypothesized model (Model 1), in addition to the theoretical reasoning, we have checked the model’s fit by testing the model in the opposite direction (Model 2). The fit indices of the reversed model (Model 2) are also reported in Table 3A, thereby indicating that the hypothesized model (Model 1) is a better fit in comparison to the reversed model.

**Table 3A:** Measurement model fit indices of study variables.

Construct	CMIN/ DF (<5)	RMR (<0.08)	TLI (>0.90)	CFI (>0.90)	RMSEA (<0.05)	GFI (>0.90)
Psychological capital	1.934	0.024	0.992	0.994	0.047	0.964
Perceived gender equity	2.969	0.049	0.971	0.975	0.068	0.9
Subjective well-being	2.975	0.063	0.963	0.968	0.068	0.916
Commitment	2.196	0.021	0.993	0.997	0.053	0.99
Social support	1.928	0.031	0.993	0.997	0.046	0.989

**Table 3B:** Model fit indices.

Model	CMIN/DF (<5)	RMR (<0.08)	TLI (>0.90)	CFI (>0.90)	RMSEA (<0.05)	GFI (>0.90)
Model 1	1.985	0.024	0.951	0.963	0.047	0.964
Model 2	2.415	0.173	0.931	0.935	0.057	0.765

The Cronbach’s alpha values were calculated for each measure to determine their reliability on the study sample. All the values of Cronbach’s alphas were > 0.70, which is statistically significant (Table 4A). The convergent validity was established, as each of the constructs reported a value greater than 0.50 for the average variance extracted (AVE) (Russell, 1978). As presented in Table 4B, discriminant validity was established as the following three conditions were met: maximum shared variance (MSV) was greater than the average shared variance (ASV), MSV was less than ASV and ASV less than AVE, and the root of average variance extracted for each construct was greater than the inter-construct correlation (Lucas et al., 1996).

**Table 4A:** Reliability, average variance extracted (AVE), maximum shared variance (MSV), and average variance shared (ASV).

Construct	Reliability	AVE	MSV	ASV
Psychological capital	0.995	0.981	0.57	0.322
Perceived gender equity	0.989	0.759	0.536	0.297
Career success	0.995	0.991	0.294	0.226
Subjective well being	0.929	0.868	0.437	0.2
Commitment	0.944	0.737	0.57	0.335
Social support	0.933	0.7	0.536	0.18

**Table 4B:** Comparison of square root of average variance extracted (AVE) and inter-construct correlations.

Construct	Square root of AVE	Psychological capital	Social well being	Perceived gender equity	Commitment	Social support
Psychological capital	0.99		0.66	0.48	0.75	0.27
Well-being	0.925	0.66		0.12	0.56	−0.007
Perceived gender equity	0.885	0.486	0.12		0.65	0.73
Commitment	0.858	0.755	0.56	0.65		0.35
Social support	0.839	0.274	−0.007	0.73	0.35	

Two tests, namely Harman’s single factor test (Harman, 1976; Podsakoff et al., 2003) and common latent factor (CLF) analysis (Lindell and Whitney, 2001), were employed to check for common method bias, as all the participants responded through a single survey instrument. For Harman’s single factor test, an exploratory factor analysis was performed to check whether a single factor could explain the majority of the variance in the data. The test results confirmed that a single factor was not accounting for the large variance, thereby confirming that common method bias was not present (Konrad and Linnehan, 1995). Also, CLF analysis was carried out to test for common method bias. CLF captured the common variance among all observed variables. A latent factor was added to the existing CFA model and then connected to all observed items in the model. Then the standardized regression weights of the constrained model were compared to the standardized regression weights of the unconstrained model. The difference in the path coefficient between the constrained and unconstrained model was less than 0.20 for each of the paths (Lindell and Whitney, 2001); therefore, we conclude that the issue of common method bias is absent.

### Path Analysis

**FIGURE 2:**
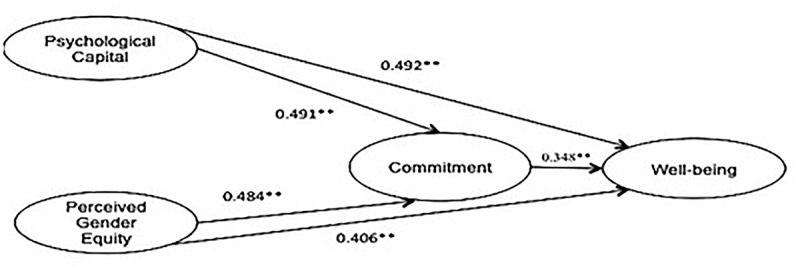
Path model for PsyCap and PGE mediation of commitment in employee well-being. The path model is for full sample. Fit indices for the model: CMIN/df = 1.984, GFI = 0.912, TLI-0.899, RMSEA = 0.028, NFI-0.823. ^∗∗^*p* < 0.05.

The structural path model was developed on the basis of the hypotheses generated. The hypotheses and the relations between the variables were tested on the basis of the regression estimates for the accepted structural path model as reported in Table 5. The model was assessed using various fit indices (reported in Figure 2). All the hypotheses were tested for the full sample, male and female managers separately, and a comparison using multi-group analysis to see if there are any significant differences between men and women for each of the relations was conducted. The structural model for the full sample is shown in Figure 2.

**Table 5:** Hypothesis testing through regression estimates.

Path from	Path to	Full			Female			Male		
		Estimate	SE	CR	Estimate	SE	CR	Estimate	SE	CR
PsyCap	Commitment	0.491^∗∗^	0.054	9.04	0.576^∗∗^	0.05	10.33	0.514^∗∗^	0.048	10.63
PGE	Commitment	0.484^∗∗^	0.053	9.22	0.465^∗∗^	0.05	8.81	0.26^∗∗^	0.045	5.761
Commitment	Well-being	0.348^∗∗^	0.061	5.68	0.33^∗∗^	0.06	4.87	0.355^∗∗^	0.092	3.878
PGE	Well-being	−0.406^∗∗^	0.049	−8.35	−0.329^∗∗^	0.05	−6.39	−0.295^∗∗^	0.055	−5.401
PsyCap	Well-being	0.492^∗∗^	0.052	9.38	0.504^∗∗^	0.06	8.37	0.338^∗∗^	0.067	5.046

*^∗∗^p < 0.05.*

As predicted in hypothesis 1(a), a significant positive impact of PsyCap on well-being is observed for the full sample (β = 0.492, *p* < 0.05) for both men (β = 0.338, *p* < 0.05) and women (β = 0.504, *p* < 0.05). It can also be seen that a greater impact of PsyCap on well-being is noted in the case of women compared to men. Furthermore, the comparison of the impact of PsyCap on well-being [Hypotheses 1(b) and 1(c)] indicates that this difference is statistically significant (CR = 3.21, *p* < 0.05). As posited in Hypothesis 2(a), a significant positive impact of PGE on well-being (β = 0.406, *p* < 0.05) is observed for the full sample for both men (β = 0.295, *p* < 0.05) and women (β = 0.429, *p* < 0.05). This shows that the impact of PGE on well-being is higher in the case of women as compared to men. The comparison of the impact of PGE on well-being between men and women [Hypotheses 2(b) and 2(c)] indicate that this difference is statistically significant (CR = 3.450, *p* < 0.05).

We checked the role of commitment as a mediator (Hypotheses 3 and 4) by adopting the most recent and acceptable method of bootstrapping as suggested by Preacher and Hayes (2008). We carried out the bootstrapping with a plugin created by Hayes (2012) to test the indirect effect between the two predictor variables (PsyCap and PGE) and the outcome variable (well-being) through the mediator (commitment). Table 6A presents the mediation analysis of commitment between PsyCap and well-being using 1,000 bootstrap simulations for the total sample. The results reveal that the total effect between PsyCap and well-being was positively associated (*B* = 0.57, SE = 0.03, *p* < 0.001 [95% CI −0.51,0.63]). Furthermore, PsyCap exerted an indirect effect on well-being through commitment (*B* = 0.16, SE = 0.029, *p* < 0.001 [95% bias-corrected bootstrap CI 0.11,0.22]), while the direct effect between PsyCap and well-being was also significant (*B* = 0.411, SE = 0.04, *p* < 0.001 [95% CI −0.33,0.486]). This implies that commitment partially mediates the relationship between PsyCap and well-being. Also, the indirect effect is statistically different from zero, as revealed by a 95% bias-corrected bootstrap confidence interval, which makes us accept Hypothesis 3. Similarly, for Hypothesis 4, we can infer from Table 6B, presenting mediation of commitment between PGE and well-being, that the indirect and direct effects are both significant at *p* < 0.001, indicating partial mediation.

**Table 6A:** Mediation analysis of commitment between PsyCap and Well-being (total sample).

Path	B	SE	*t*	LLCI	ULCI
c path (total effect)	0.579^∗∗∗^	0.038	15.237	0.51	0.637
c’ path (direct effect)	0.411^∗∗∗^	0.046	8.935	0.333	0.486
Indirect effect	0.168^∗∗∗^	0.029	5.793	0.124	0.223

*Number of bootstrap samples for bias corrected bootstrap confidence intervals: 2000, for all confidence intervals: 95. ^∗∗∗^p < 0.001.*

**Table 6B:** Mediation analysis of commitment between PGE and Well-being (total sample).

	B	SE	*t*	LLCI	ULCI
c path (PGE → SWB) total	0.177^∗∗∗^	0.041	4.317	0.224	0.117
c’ path (PGE → SWB) direct	0.307^∗∗∗^	0.045	6.822	0.38	0.232
Indirect effect	0.130^∗∗∗^	0.026	5.000	0.090	0.175

*Number of bootstrap samples for bias corrected bootstrap confidence intervals: 2000, for all confidence intervals: 95. ^∗∗∗^p < 0.001.*

**FIGURE 3:**
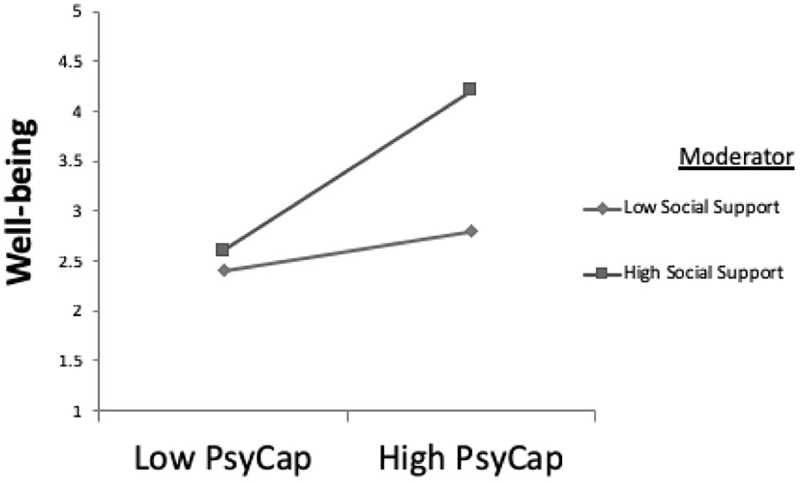
The moderating effect of social support on PsyCap–Well-being relationship (total sample).

**FIGURE 4:**
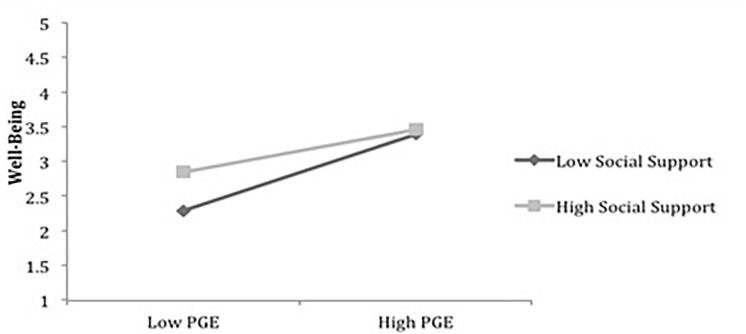
The moderating effect of social support on PGE–Well-being relationship (total sample).

To test the moderation effect in Hypotheses 5(a), 5(b), and 5(c) (social supports moderates the relationship between PscyCap and well-being), we standardized PsyCap (ZPSYCAP) and social support (ZSS) and created an interaction term, ZSS_X_ ZPSYCAP. From Table 7A, we observe that ZPSYCAP and ZSS, as well as their interaction, have a significant impact on well-being, indicating that moderation occurs for the full sample. Following a similar procedure for the data for men and women, it is inferred that social support positively moderates the relationship between PsyCap and well-being. For a comparison between men and women, with respect to moderation effects, evaluating all the paths against 2.00 as the critical level of comparison, it is seen that none of the coefficients differ significantly for men and women (Table 7B). Similarly, testing for Hypothesis 6 (moderating effect of social support between PGE and well-being), we standardized PGE (ZPGE) and social support (ZSS) and created an interaction term ZSS_X_ZPGE. From Table 7A, it can be observed that both ZPGE and ZSS are significant while their interaction has no significant impact on SWB, indicating that moderation does not occur for the full sample. Similarly, for male and female samples, we posit that social support does not moderate the relationship between PGE and well-being. For comparing this between men and women [Hypotheses 6(b) and 6(c)] with 2.00 as the critical level of comparison, we observe that none of the coefficients differ significantly for men and women (Table 7C). Furthermore, we plotted the slopes to infer the strength of the moderation effect in the full sample of men and women. The slopes plotted for the full sample are shown in Figure 3, 4.

**Table 7A:** Moderation analysis: path estimates.

		Full sample			Women			Men		
Path from	Path to	Estimate	SE	CR	Estimate	SE	CR	Estimate	SE	CR
ZPSYCAP	Well-being	0.827^∗∗∗^	0.04	20.56	0.899^∗∗∗^	0.05	18.098	0.754^∗∗∗^	0.063	11.90
ZSS	Well-being	0.20^∗∗∗^	0.04	−5.22	−0.197^∗∗∗^	0.047	−4.188	−0.216^∗∗∗^	0.063	−3.43
ZSS_X_ZPSYCAP	Well-being	0.28^∗∗∗^	0.037	7.79	0.308^∗∗∗^	0.044	6.949	0.249^∗∗∗^	0.06	4.15
ZPGE	Well-being	0.30^∗∗∗^	0.1	3.01	0.394^∗∗∗^	0.152	2.599	0.23^∗∗∗^	0.132	1.74
ZSS	Well-being	−0.25^∗∗∗^	0.08	−3.14	−0.341^∗∗∗^	0.123	−2.762	−0.216^∗∗∗^	0.109	−1.98
ZSS_X_ZPGE	Well-being	−0.042	0.08	−0.47	0.102	0.139	0.733	−0.147	0.115	−1.28

*ZPSYCAP is the standardized PsyCap variable, ZSS is standardized social support variable, ZSS_X_ZPSYCAP is the interaction term, ZPGE is standardized PGE and ZSS_X_ZPGE is the interaction term. ^∗∗∗^p < 0.05.*

**Table 7B:** Moderation analysis: critical ratio for differences (comparison between men and women for PsyCap).

	W1	W2	W3	M1	M2	M3
W1	0					
W2	3.403	0				
W3	16.109	13.305	0			
M1	2.326	0.402	11.125	0		
M2	4.251	1.547	9.189	1.519	0	
M3	11.915	9.284	1.639	10.244	7.113	0

*W1 denotes the path Well-being < ZPSYCAP for women, W2 denotes the path well-being < ZSS for women, W3 denotes the path well-being < ZSS_X_ZPSYCAP for women, M1 denotes the path Well-being < ZPSYCAP for men, M2 denotes the path well-being < ZSS for men, M3 denotes the path well-being < ZSS_X_ZPSYCAP.*

**Table 7C:** Moderation analysis: critical ratio for differences (comparison between men and women for PGE).

	W1	W2	W3	M1	M2	M3
W1	0					
W2	−1.347	0				
W3	−4.35	−0.746	0			
M1	−0.443	1.478	2.248	0		
M2	−1.852	0.098	1.018	−1.081	0	
M3	−3.807	−2.136	−1.107	−5.905	−1.935	0

*W1 denotes the path Well-being < ZPGE for women, W2 denotes the path well-being < ZSS for women, W3 denotes the path well-being < ZSS_X_ZPGE for women, M1 denotes the path Well-being < ZPGE for men, M2 denotes the path well-being < ZSS for men, M3 denotes the path well-being < ZSS_X_ZPGE.*

## Discussion

Our findings provide insights that enrich the existing theory and research and add new dimensions to the literature of improving well-being, especially in women. Our study investigated and found evidence for the positive impact of the positive psychological resource of PsyCap and perceived gender equity on employees’ well-being. The study’s support for mediation of commitment provides for a convincing reason for organizations to invest in enhancing the well-being of their employees. We elaborate on the theoretical and practical implications of our findings for the PsyCap, PGE, and well-being literatures in the following section.

### Theoretical Implications

The study’s theoretical implications are diverse and many. The studies examining well-being in women are few and far between. These studies have examined the physical health, burnout, stress, or psychological well-being in a few cases. Subjective well-being, which is currently the dominant conception of well-being in psychological literature (David et al., 2013) and is of importance to the organizations as well (Cameron and Caza, 2013), has been seldom studied. This study, therefore, extends the literature on subjective well-being by being possibly the first study to examine the factors leading to subjective well-being of female managers in the service industry in India.

Through the acceptance of Hypotheses 1(a), 1(b), and 1(c), it implies that PsyCap leads to well-being in both male and female managers. It further strengthens the conservation of resources theory and employee well-being (Hobfoll, 2002; Wright and Hobfoll, 2004). This is one of the first studies emphasizing the importance positive PsyCap for working women to improve their well-being. The study further strengthens the theory of the relationship between positive resources and well-being, more in the case of women as indicated by these results. There is no prior study that has investigated the role of PsyCap in female employees’ well-being, given their differential circumstances as compared to men. Hence, this is possibly the first empirical study investigating and establishing the positive relationship between PsyCap and the well-being of female managers.

The acceptance of Hypotheses 2(a), 2(b), and 2(c) furthers the empirical evidence for the feeling of equity being an important factor contributing to well-being and stress reduction (David et al., 2013) for both male and female managers; at the same time, it is found different in affecting the well-being of men vs. women, with the effect being greater on women as hypothesized in Hypothesis 2(c). Except for Sharma and Sharma (2015), no study has investigated the relationship between perceived gender equity and women’s well-being. However, the conceptualization of the outcome variable of well-being is different in the aforementioned study compared to this investigation.

The acceptance of Hypotheses 3 and 4 confirms the mediating role of commitment in the relations of PsyCap and perceived gender equity (the independent variables) to employee well-being (the dependent variable). This indicates that PsyCap and perception of gender equity at the workplace can result in enhanced commitment, which in turn, can lead to the subjective well-being of employees.

The acceptance of Hypotheses 5(a), 5(b), and 5(c) implies that social support contributes significantly and strengthens the relationship when combined with PsyCap to impact the well-being of both men and women. However, it is seen that there is no significant difference between the moderating effect that social support has on men vs. women. This finding is not as proposed by our initial assumption of Hypothesis 5(b), which stated that social support would strengthen the relationship between PsyCap and well-being, considering the multiple responsibilities women hold at work and home, and that, therefore, support from family and peers would contribute more to women’s well-being. We further explored causes of this finding in the study. Our sample data indicate that the marital status of the males and the working status of their wives might be a possible cause of no difference in the moderating role of social support. 90% of the men in the sample were married, 81% of the married men had wives who were employed full time, and 74% had children. A possible inference could be that dual-career couples realize the importance of providing equal social support to cope with work/life challenges (Kaur and Kumar, 2017) and share the responsibilities of taking care of children and/or ageing parents (Burke, 2017). Also, a few studies suggest that as the male and female work and family roles change over time, some of the preexisting gender differences might become less pronounced, with men and women contributing more equally at home and at work (Drummond et al., 2017).

Hypotheses 6(a), 6(b), and 6(c) were not supported for the full sample of men as well as women. This implies that social support does not make any difference to the relationship between PGE and well-being, suggesting that the perception of having a gender-equitable work environment supersedes the social support that men and women may receive from family, supervisors, or peers. Thereby, this emphasizes the importance of PGE in the workplace over social support to ensure the well-being of both male and female employees. Furthermore, no difference was found between men and women for the moderating role of social support between PGE and well-being as hypothesized in Hypotheses 6(b) and 6(c).

### Practical Implications

The study establishes the importance of PsyCap’s role in female managers’ well-being. PsyCap, being a state-like resource (Luthans et al., 2007, 2008), makes it open to development through interventions. Therefore, providing organizations with an opportunity to facilitate training for women to develop PsyCap to ensure their well-being, in turn, allows them to better contribute to the organization. Earlier research (Luthans et al., 2006) suggested the psychological capital intervention (PCI) for organizations to implement to increase the PsyCap of individuals. This has also been proven to significantly increase (around 2%) the level of individual psychological capital. Furthermore, implementing short face-to-face training and web-based training interventions has been proposed as an operationalization of the PCI model that also minimizes the time and costs related to its implementation and facilitates the development of individual PsyCap (Luthans et al., 2008, 2010).

The positive relationship between PGE and well-being draws the organization’s attention to an important gap pointed out by Burgess et al. (2007): that policies involving the reconciliation of work and care, temporary withdrawal from work, leave arrangement, and professional support are not the only factors that ensure that female employees balance work and life. Equally necessary is to provide an environment to avail of such policies. Therefore, it compels the organizations to think and act in totality of all three (environment, policy and practice), hence the study would have policy and implementation implications for organizations.

The acceptance of commitment as a mediator further strengthens organizations’ interest in developing employees’ PsyCap and creating a gender-equitable workplace through its policies, the implementation of the same, and the environment, to ensure the policies are executed in the right spirit. This would not only affect the well-being of employees, but also employees’ affective commitment toward the organization, which positively affects their performance at work (Vandenberghe et al., 2004).

## Strengths, Limitations, and Future Research

The study yielded high response rates from middle-level managers, particularly women in the service sector, using the personal survey method. We also acknowledge limitations. The cross-sectional design of our survey limits our claims of causality in the tested model. However, our findings were consistent with and informed by existing extant theoretical and empirical research, and our findings provide further confirmation of this. Longitudinal studies can be conducted in the future to address this issue. The study’s self-report questionnaire and single-source design may raise concerns about common-method bias. While our statistical tests negate the possibility of this, we acknowledge that there could be a possibility of some bias effect. Future studies could use multi-source research designs to handle this limitation. We have used the positive psychological construct of PsyCap, as we wanted to focus on the positive or facilitating factors that could contribute to the well-being of women. However, we acknowledge the limitations of positive psychology, one of them being “culturally restrictive” as put forward by Fineman (2006), where he states that positive emotions in positive psychology tend to drift toward the North American cultural norms of celebrating individualism, optimism, and self-confidence. Future studies may bring in the cultural nuances affecting such perceptions. While we checked for perceived gender equity’s impact on individual outcomes (well-being) and organizational outcome (commitment), future studies may focus on more organizational outcomes. The sensitivity around the subject of gender equity led us to include an anonymity clause, which limits the study’s possibility of longitudinal inquiry into the subject. Future studies may look into a longitudinal design to get deeper insights. While the study assesses the mediating and moderating mechanisms operating between the positive relationship between PsyCap and well-being, and PGE and well-being, future studies may look at a qualitative inquiry into uncovering these and other operating mechanisms. The study’s sample includes only the private sector; future studies may look at the viability and insights from a similar study in public-sector organizations. Cross-cultural studies would be insightful in identifying the determinants of women’s well-being in other geographies. Our study was limited to middle- and senior-level female managers; future studies can focus on entry-level women in organizations, which can reveal more factors related to women’s well-being. While the current study explores the role of PGE at work, an equally important factor determining women’s well-being could be a similar construct like PGE at home, evaluating the equity perception at home as well.

## Conclusion

The importance of employees’ well-being to organizations, the scarcity of female talent, the adverse effects of juggling between multiple roles on working women’s well-being and the absence of scholarly work in the area of improving working women’s well-being provided impetus for this study. The study contributes to the growing body of research on well-being by unearthing ways in which PsyCap and PGE enhance employees’ well-being. The findings of the study will particularly contribute to literature concerning the facilitation of women’s well-being in organizations. Furthermore, it would help organizations to devise ways of dealing with employee absence, turnover, or declining productivity due to reduced well-being (David et al., 2012). PsyCap as a resource that can be developed (Luthans et al., 2007) can be closely looked at by organizations to be nurtured in female employees through training interventions.

The study contributes to the literature on well-being by establishing a positive relationship between perceived gender equity and the subjective well-being of employees and female employees in particular. It determines the role of a woman’s psychological capital in enhancing their well-being and opens vistas for gender-based research. The positive influence of psychological capital and perceived gender equity on well-being occurs through the mediating mechanism of commitment, which is an important finding to encourage organizations to invest in employees’ well-being. In an era in which the current generation’s employees are entrusted with childcare and elder care (Burke, 2017), the role of social support cannot be undermined. The study also investigates the role of social support in moderating the relationship between PsyCap, PGE, and well-being.

## Ethics Statement

This manuscript is based on part of the doctoral work of SC, who received a fellowship at the Management Development Institute in Gurgaon, India. It has been developed under the guidance of RS. Approval from the ethics committee could not be solicited for the present work, as the Management Development Institute does not provide this service for doctoral students. Informed consent was obtained from all study participants (who are adults) prior to conducting this research. From the cover pages of the questionnaires and the study’s introductory talk, all participants were informed of the research purposes, their freedom of participating in and quitting the research, and the assurance of confidentiality. Also, it was reiterated to them that participation in the study was absolutely voluntary and that they could discontinue participation at any time. This research pertains to the social sciences, which do not require the approval of the Ethics Committee at the institutional or the national level.

## Author Contributions

SC and RS contributed to the design, implementation, and analysis of the results of the study, and to the writing of the manuscript.

## Conflict of Interest Statement

The authors declare that the research was conducted in the absence of any commercial or financial relationships that could be construed as a potential conflict of interest.

## Footnotes

1 https://www.greatplacetowork.com/resources/blog/15-practice-areas-critical-to-achieving-a-great-workplace (accessed on July 20, 2016).
